# New Fluorene Derivatives from *Dendrobium gibsonii* and Their *α*-Glucosidase Inhibitory Activity

**DOI:** 10.3390/molecules25214931

**Published:** 2020-10-25

**Authors:** May Thazin Thant, Nutputsorn Chatsumpun, Wanwimon Mekboonsonglarp, Boonchoo Sritularak, Kittisak Likhitwitayawuid

**Affiliations:** 1 Department of Pharmacognosy and Pharmaceutical Botany, Faculty of Pharmaceutical Sciences, Chulalongkorn University, Bangkok 10330, Thailand; maythazinthant@mohs.edu.mm (M.T.T.); Kittisak.L@chula.ac.th (K.L.); 2 Department of Pharmacognosy, Faculty of Pharmacy, Mahidol University, Bangkok 10400, Thailand; nutputsorn.cha@mahidol.ac.th; 3 Scientific and Technological Research Equipment Centre, Chulalongkorn University, Bangkok 10330, Thailand; wanwimon.m@chula.ac.th; 4 Natural Products for Ageing and Chronic Diseases Research Unit, Faculty of Pharmaceutical Sciences, Chulalongkorn University, Bangkok 10330, Thailand

**Keywords:** *Dendrobium gibsonii*, Orchidaceae, fluorene derivative, dihydrophenanthrenes, α-glucosidase inhibitory activity

## Abstract

Two new compounds, dihydrodengibsinin (**1**) and dendrogibsol (**2**), were isolated from the whole plant of *Dendrobium gibsonii*, together with seven known compounds (**3**–**9**). The structures of the new compounds were elucidated by their spectroscopic data. All these isolates were evaluated for their α-glucosidase inhibitory activities. Dendrogibsol (**2**) and lusianthridin (**7**) showed strong α-glucosidase inhibitory activity when compared with acarbose. An enzyme kinetic study revealed that dendrogibsol (**2**) is a noncompetitive inhibitor of α-glucosidase.

## 1. Introduction

Diabetes is a metabolic disease associated with chronic hyperglycemia due to deficiency in insulin secretion or action [1]. The prevalence of diabetes has been increasing all over the world. Around 8.8% of the world’s adult population suffered from diabetes in 2017, and it is estimated that the number will rise to 9.9% by 2045 [2]. Many diabetic patients suffer from chronic complications such as nephropathy, neuropathy, retinopathy and macrovascular problems, which are the major causes of morbidity and mortality. About 90% of all diabetic patients are caused by type II diabetes [3].

α-Glucosidase is one of the key enzymes involved in carbohydrate metabolism and is essential for maintaining normal physiological functions [4]. It has been considered a suitable model for observing the action of nutraceuticals on type II diabetes [5]. α-Glucosidase inhibitor (α-GI) drugs, given alone or combination with other oral antidiabetic agents, have been used for the treatment of type II diabetes [6]. Acarbose and miglitol are examples of α-GIs; they decrease postprandial hyperglycemia by retarding the absorption of glucose in the intestine [7]. However, these drugs have several side effects, including diarrhea, flatulence, abdominal pain and liver damage [8]. Thus, new α-GI drugs with less adverse effects are still needed. α-Glucosidase enzymes obtained from yeast, rat intestine and mouse intestine have been used as screening tools for identifying potential α-GI agents [9].

A large number of α-GIs have been reported from natural sources [10]. Several α-GIs of plant origin appear to be more potent and safer than their synthetic counterparts [11]. *Dendrobium*, a major genus in the family Orchidaceae, is widely distributed throughout Asia and Australia, with about 150 species that have been identified in Thailand [12]. Several plants in this genus are widely known in traditional Chinese medicine as a tonic to increase body fluid and reduce fever [13]. They can be also used to cure kidney, lung and stomach diseases; red tongue; inflammation; dry mouth; hyperglycemia; and diabetes [14]. In Thailand, some species of *Dendrobium*, for example, *D. cumulatum*, *D. draconis*, *D. indivisum*, *D. trigonopus* and *D. leonis*, have also been used in traditional medicine [15]. However, in spite of their numerous medicinal reputations, only a few species of *Dendrobium* have been investigated so far [16]. Previous reports on the *Dendrobium* genus have revealed the presence of phenanthrenes, bibenzyls, sesquiterpenoids, alkaloids and polysaccharides and disclosed many interesting biological activities, such as antitumor, anti-inflammatory and platelet antiaggregation activities [17].

*Dendrobium gibsonii* Paxton, known as Ueang Kham Ta in Thai, is an epiphytic orchid with slender stems, lanceolate leaves and orange to yellow colored flowers [18,19]. It is found in India, Nepal, Bhutan, Myanmar, Thailand, China and Vietnam. An earlier phytochemical study on this plant uncovered two fluorenone derivatives, namely dengibsin and dengibsinin [20,21]. As a part of our continuing studies on α-glucosidase inhibitors from *Dendrobium* spp. [22,23], a MeOH extract prepared from the whole plant of *D. gibsonii* was evaluated and found to exhibit significant inhibitory activity against the enzyme (78.7 ± 3.2% inhibition at 100 µg/mL). In this communication, we report the isolation and structural characterization of the active principles of this plant.

## 2. Results and Discussion

### 2.1. Structural Characterization

**Figure 1:**
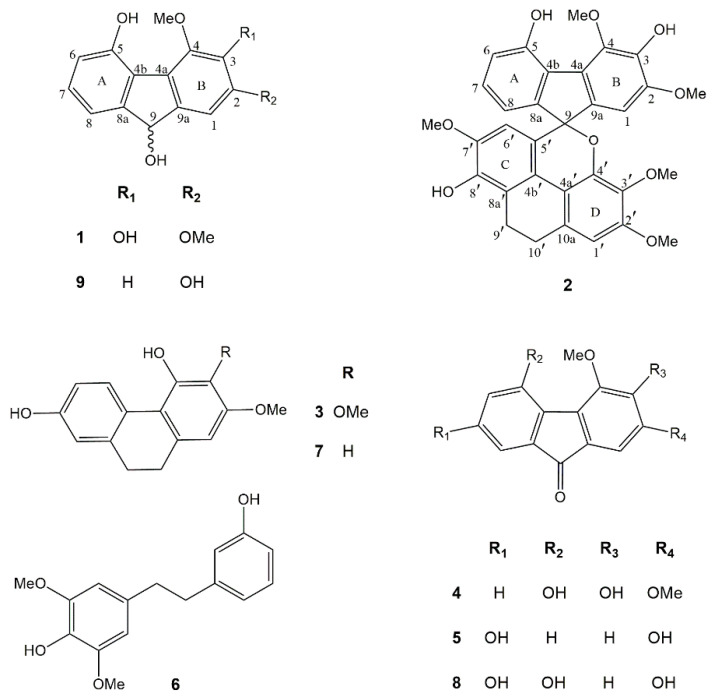
Chemical structures of compounds **1**–**9** isolated from *Dendrobium gibsonii*.

Phytochemical investigation of the EtOAc extract of *D. gibsonii* resulted in the isolation of two new compounds named dihydrodengibsinin (**1**) and dendrogibsol (**2**), together with seven known compounds, namely ephemeranthol A (**3**) [24], dengibsinin (**4**) [21], nobilone (**5**) [25], aloifol I (**6**) [26], lusianthridin (**7**) [27], denchrysan A (**8**) [28] and 4-methoxy-9*H*-fluorene-2,5,9-triol (**9**) [29] (Figure 1). The known compounds (**3**–**9**) were identified through comparison of their spectroscopic data with literature values. The structures of the new compounds (**1** and **2**) were elucidated by analysis of their NMR and HRMS data.

Compound **1** was obtained as a brownish-white amorphous solid. The molecular formula C_15_H_14_O_5_ was analyzed from its [M − H]^−^ at *m*/*z* 273.0764 (calcd. for C_15_H_13_O_5_ 273.0763). The IR spectrum showed absorption bands for hydroxyl (3420 cm^−1^) and aromatic (2925, 1618 cm^−1^) functionalities. The UV spectrum exhibited absorption peaks at 220, 255 and 300 nm, indicating a fluorene structure [30]. This was supported by the presence of twelve aromatic carbons and one oxygenated methine carbon of C-9 (δ 74.5), which correlated to the proton at δ 5.38 (1H, d, *J* = 7.8 Hz, H-9) in the HSQC spectrum (Table 1). The HO-9 proton at δ 4.57 (d, *J* = 7.8 Hz) displayed two-bond HMBC correlation with C-9. The ^1^H-NMR spectrum of **1** showed four aromatic proton signals at δ 6.77–7.13 and signals for two methoxyl groups at δ 3.93 (3H, s, MeO-2) and δ 4.12 (3H, s, MeO-4). On ring A, the ^1^H-NMR spectrum exhibited three aromatic protons with *ortho*-coupling at δ 6.77 (1H, d, *J* = 7.5 Hz, H-6), 7.05 (1H, d, *J* = 7.5 Hz, H-8) and 7.13 (1H, t, *J* = 7.5 Hz, H-7). The assignment of H-8 was based on its HMBC correlations with C-9. The HO-5 proton at δ 9.44 (s) showed correlation with C-5 (δ 151.1) and C-6 (δ 116.1) in the HMBC spectrum. On ring B, the singlet proton signal δ 7.10 was assigned to H-1 from its HMBC correlation with C-9. The first methoxyl (δ 3.93) was located at C-2 and the second methoxyl (δ 4.12) was at C-4, as supported by their NOESY correlations with H-1 and HO-5, respectively. Based on the above spectral data, compound **1** was characterized as 2,4-dimethoxy-9*H*-fluorene-3,5,9-triol and given the trivial name dihydrodengibsinin. Prior to this study, the natural occurrence of **1** was not known. This compound, however, was earlier synthesized by reduction of the corresponding fluorenone dengibsinin [20,21].

Compound **2** was obtained as a brownish amorphous solid. The molecular formula C_32_H_28_O_9_ was deduced from its [M + H]^+^ at *m*/*z* 557.1825 (calcd. for C_32_H_29_O_9_ 557.1811). The IR spectrum exhibited absorption bands at 3334 (hydroxyl), 2930, 1607 (benzene ring), 1485 (methylene) and 1236 (ether) cm^−1^. The UV spectrum exhibited absorption peaks at 260, 310 and 325 nm. Comparison of ^1^H and ^13^C-NMR data of **2** with **1** (Table 1) suggested that **2** was an adduct of fluorene and dihydrophenanthrene structures. Compound **2** showed several ^1^H-NMR resonances similar to those of the fluorene **1**, representing four aromatic protons at δ 6.65 (1H, dd, *J* = 8.0, 1.0 Hz, H-8), 6.76 (1H, dd, *J* = 8.0, 1.0 Hz, H-6), 6.85 (1H, s, H-1) and 6.93 (1H, t, *J* = 8.0 Hz, H-7) and two methoxyl groups at C-2 (δ 3.77, 3H, s,) and C-4 (δ 4.18, 3H, s). The presence of a dihydrophenanthrene unit in **2** was deduced from the characteristic signals for 2 methylene carbons at δ 20.9 (C-9′) and 26.9 (C-10′) in addition to 12 aromatic carbon resonances. In the ^1^H-NMR spectrum, the dihydrophenanthrene unit displayed two aromatic proton singlets at δ 6.04 (1H, s, H-6′) and 6.61 (1H, s, H-1′), and three methoxyl groups at δ 3.37 (3H, s, MeO-3′), 3.54 (3H, s, MeO-7′) and 3.82 (3H, s, MeO-2′). The assignment of H-6′ of ring C was supported by its HBMC correlations with C-4b′ (δ 120.6) and C-8′ (δ 143.4). On ring C, the first methoxy group should be placed at C-7′ according to its NOESY correlation with H-6′. On ring D, the assignment of H-1 was deduced from its HMBC correlations with C-10′. The NOESY cross-peak between H-1′ and H_2_-10′ was also observed. The second methoxy group was located at C-2′, as supported by its NOESY correlation with H-1′. The HMBC correlations of C-3′ (δ 137.3) with H-1′ and MeO-3′ indicated the location of the third methoxy group at C-3′. Compound **2** had the fluorene moiety connected to the dihydrophenanthrene unit through a C–C linkage between C-5′ (δ 123.4) and C-9 (87.4) and ether bond between C-9 and the oxygen atom at C-4′ (δ 145.3), forming a spiro structure. This was supported by the HMBC correlations of C-9 with H-1, H-8 and H-6′. Thus, it was concluded that **2** was a fluorene–dihydrophenanthrene adduct, with the structure as shown in Figure 1, and it was given the trivial name dendrogibsol. It is the first representative of this class of dimeric compounds.

**Figure 2:**
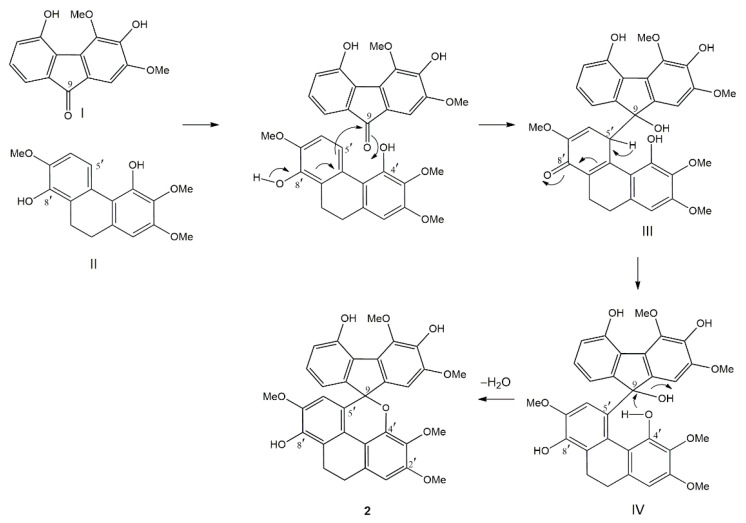
Possible biogenesis of **2**.

The biogenesis of the unprecedented fluorene–dihydrophenanthrene adduct (**2**) is proposed to occur as shown in Figure 2. The coupling reaction is initiated by the nucleophilic attack from C-5′ of the dihydrophenanthrene unit (II) onto the keto carbon (C-9) of the fluorenone (I) to give a quinone-like structure (III). This structure subsequently isomerizes to form intermediate IV. Finally, the nucleophilic attack by the oxygen of the OH-4′ group of the dihydrophenanthrene unit on the carbinol carbon (C-9) of the fluorene part, with concomitant loss of H_2_O, generates compound **2**.

### 2.2. *α*-Glucosidase Inhibitory Activity

All the isolated compounds (**1**–**9**) were evaluated for their α-glucosidase inhibitory activities. In this study, each compound was initially tested at 100 μg/mL. Half-maximal inhibitory concentration (IC_50_) was determined if the compound showed more than 50% inhibition of the enzyme. Acarbose was used as the positive control. Dendrogibsol (**2**) and lusianthridin (**7**) showed potent α-glucosidase inhibitory activities with IC_50_ values of 19.8 ± 0.9 μM and 185.4 ± 6.9 μM, respectively, when compared with acarbose (IC_50_ 514.4 ± 9.2 μM). The other compounds were devoid of activity.

**Figure 3:**
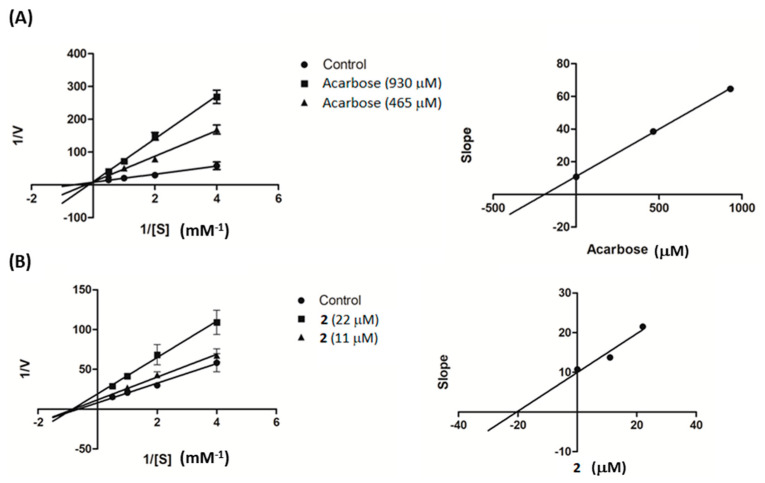
Lineweaver–Burk plots of (**A**) acarbose and (**B**) compound **2**. The secondary plot of each compound is on the right.

Further investigation was conducted on compound **2** to study its kinetic properties with regard to the enzyme α-glucosidase using varying concentrations of the substrate. From Lineweaver–Burk plots in Figure 3A, it can be seen that acarbose inhibited α-glucosidase in a competitive manner. When the acarbose concentration was increased, the K*_m_* decreased from 6.74 to 1.55 mM while the V*_max_* value (0.11 ∆OD/min) was unaffected. On the other hand, compound **2** was found to be a noncompetitive inhibitor of α-glucosidase, with decreasing V*_max_* from 0.12 to 0.052 ∆OD/min and unchanging K*_m_* (1.55 mM), as illustrated in Figure 3B. The generated secondary plots for compound **2** and acarbose revealed that the K*_i_* value of **2** (20.38 µM) was much less than that of acarbose (190.57 µM), as shown in Figure 3 and summarized in Table 2.

## 3. Materials and Methods

### 3.1. General Experimental Procedures

UV spectra were measured by a Milton Roy Spectronic 3000 Array spectrophotometer (Rochester, Monroe, NY, USA), and IR spectra by were measured by a PerkinElmer FT-IR 1760X spectrophotometer (Boston, MA, USA). Mass spectra were obtained from a Bruker MicroTOF mass spectrometer (ESI-MS) (Billerica, MA, USA). NMR spectra were recorded on a Bruker Avance DPX-300FT NMR spectrometer or a Bruker Avance III HD 500 NMR spectrometer (Billerica, MA, USA). Microtiter plate reading was analyzed by a Biochom EZ Read 400 microplate reader (Cambridge, UK). Optical rotation was measured by a PerkinElmer Polarimeter 341 (Boston, MA, USA). Vacuum liquid column chromatography (VLC) and column chromatography (CC) were performed on silica gel 60 (Merck, Kieselgel 60, 70–320 mesh), silica gel 60 (Merck, Kieselgel 60, 230–400 mesh) (Darmstadt, Germany) and Sephadex LH-20 (25–100 μm, Pharmacia Fine Chemical Co. Ltd.) (Piscataway, NJ, USA). Yeast α-glucosidase enzyme and *p*-nitrophenol-α-d-glucopyranoside were purchased from Sigma Chemical, Inc. (St. Louis, MO, USA), and acarbose was obtained from Fluka Chemical (Buchs, Switzerland).

### 3.2. Plant Material

The whole plant of *D. gibsonii* was purchased from Chatuchak market, Bangkok, in February 2018. Plant identification was performed by B. Sritularak. A voucher specimen (BS-DG-022561) has been deposited at the Department of Pharmacognosy and Pharmaceutical Botany, Faculty of Pharmaceutical Sciences, Chulalongkorn University (Bangkok, Thailand).

### 3.3. Extraction and Isolation

The dried powder of whole-plant *D. gibsonii* (4.2 kg) was macerated with methanol (MeOH) (5 × 15 L), and a MeOH extract (371 g) was obtained. This extract was dissolved in water and then partitioned with EtOAc and BuOH to give an EtOAc extract (100 g), a BuOH extract (72 g) and an aqueous extract (95.5 g) after evaporation of the solvent. These extracts were then evaluated for their α-glucosidase inhibitory activity. Only EtOAc extract exhibited strong α-glucosidase, with 77.7 ± 1.8% inhibition at concentration 100 µg/mL, and therefore was further investigated The BuOH and aqueous extracts were devoid of activity (<50% inhibition at concentration 100 µg/mL).

The EtOAc extract was then separated by vacuum liquid chromatography (silica gel, EtOAc–dichloromethane, gradient) to give five fractions (A–E). Fraction B (8.3 g) was fractionated on a silica gel column (acetone–hexane, gradient) to give three fractions (BA–BC). Fraction BB (170 mg) was separated by Sephadex LH-20 (acetone) to yield BBA and BBB fractions. Fraction BBB (190 mg) was subjected to column chromatography (CC) (silica gel, EtOAc–hexane, gradient) to give ephemeranthol A (**3**) (18 mg) and dengibsinin (**4**) (15.7 mg). Fraction C (10.8 g) was fractionated again on a silica gel column (acetone–hexane, gradient) to give four fractions (CA–CD). Fraction CB (1.3 g) was separated by Sephadex LH-20 (acetone) to yield CBA and CBB fractions. Fraction CBA (740 mg) was subjected to CC (silica gel, EtOAc–CH_2_Cl_2_, gradient) to yield nobilone (**5**) (98 mg). Fraction CC (1 g) was separated by Sephadex LH-20 (acetone) to get three fractions (CCA, CCB and CCC). Fraction CCB (60 mg) was subjected to CC (silica gel, EtOAc–hexane, gradient) to furnish aloifol I (**6**) (11.2 mg). Fraction CCC (100 mg) was also subjected to CC (silica gel, EtOAc–hexane, gradient) to give lusianthridin (**7**) (6.2 mg) and **1** (25.3 mg). Fraction CD (805 mg) was separated by Sephadex LH-20 (acetone) to give fractions CDA and CDB. Fraction CDA (50 mg) was purified by CC (silica gel, EtOAc–dichloromethane, gradient) to yield **2** (5 mg). Fraction D (5.5 g) was further fractionated on a silica gel column (acetone–dichloromethane, gradient) to give three fractions (DA–DC). Fraction DB (1 g) was separated by Sephadex LH-20 (acetone) to yield DBA and DBB fractions. Fraction DBA (30 mg) was subjected to CC (silica gel, MeOH–toluene, gradient) to furnish denchrysan A (**8**) (14 mg). Fraction E (8.2 g) was fractionated on a silica gel column (acetone–dichloromethane, gradient) to give EA and EB fractions. 4-Methoxy-9*H*-fluorene-2,5,9-triol (**9**) (10.3 mg) was yielded after purification by Sephadex LH-20 (methanol).

Dihydrodengibsinin (**1**); brownish-white amorphous solid; ${\left[\alpha \right]}_{D}^{20}$ − 100.0 (*c* 0.01, MeOH); UV (MeOH): λ_max_ (log ε) 220 (3.82), 255 (4.02), 300 (4.24); IR (film) ν _max_: 3420, 3240, 2925, 1618, 1484, 1459, 1373, 1314, 1144, 1084, 720 cm^−1^; HR-ESI-MS: [M − H]^−^ at *m*/*z* 273.0764 (calcd. for C_15_H_13_O_5_ 273.0763).

Dendrogibsol (**2**); brownish amorphous solid; ${\left[\alpha \right]}_{D}^{20}$ + 156.0 (*c* 0.002, MeOH);UV (MeOH): λ_max_ (log ε) 260 (5.10), 310 (4.76) and 325 (4.61); IR (film) ν _max_: 3434, 2930, 2848, 1723, 1607, 1485, 1461, 1365 1303, 1282, 1236, 1198, 1092 cm^−1^; HR-ESI-MS: [M + H]^+^ at *m*/*z* 557.1825 (calcd. for C_32_H_29_O_9_ 557.1811).

### 3.4. Assay for *α*-Glucosidase Inhibitory Activity

The α-glucosidase inhibition assay was performed according to previous protocols [31]. The assay was based on the release of *p*-nitrophenol from *p*-nitrophenol-α-d-glucopyranoside (substrate). The test samples were prepared by dissolving in 50% DMSO. Two-fold serial dilution was done for IC_50_ determination of active compounds. The sample solution (10 μL) and 0.1 U/mL α-glucosidase (40 μL) in phosphate buffer (pH 6.8) were added to a 96-well plate. The mixture was preincubated at 37 °C for 10 min before adding 2 mM *p*-nitrophenol-α-d-glucopyranoside (50 μL). Then, the reaction was incubated again at 37 °C for 20 min. Finally, 1 M Na_2_CO_3_ solution (100 μL) was added to stop the reaction. The absorbance of the mixture was determined using a microplate reader at 405 nm. In this assay, acarbose was used as the positive control.

An enzyme kinetic study was conducted based on the α-glucosidase assay as mentioned above. The PNPG concentrations were varied from 0.25 to 2 mM in the absence or presence of compound **2** (11 and 22 µM) or acarbose (930 and 465 µM). The inhibition mode was determined by double-reciprocal Lineweaver–Burk plot (1/V vs. 1/[S]). In order to estimate the K*_i_* value, slopes of double-reciprocal lines were used to construct a secondary plot, and the K*_i_* was calculated from the line equation of the plot [32].

## 4. Conclusions

In this study, nine compounds were isolated from the whole plant of *Dendrobium gibsonii*, namely two new compounds—dihydrodengibsinin (**1**) and dendrogibsol (**2**)—and seven known compounds (**3**–**9**). Dendrogibsol (**2**) constituted a novel type of adduct, biogenetically derived from the coupling of a fluorenone and a dihydrophenanthrene monomer. Among the isolates, compound **2** was the most potent α-glucosidase inhibitor, followed by lusianthridin (**7**), as compared with the positive control acarbose. An enzyme kinetic study revealed that compound **2** is a noncompetitive inhibitor of α-glucosidase enzyme.

## Figures and Tables

**Table 1:** ^1^H and ^13^C-NMR spectral data of **1** and **2** in acetone-*d*_6_.

Position	1 ^a^			2 ^b^		
	δ_H_ (Multiplicity, *J* in Hz)	δ_C_	HMBC (Correlation with ^1^H)	δ_H_ (Multiplicity, *J* in Hz)	δ_C_	HMBC (Correlation with ^1^H)
1	7.10 (1H, s)	105.2	9	6.85 (1H, s)	105.6	-
2	-	148.4	1 *, HO-3, MeO-2	-	148.6	HO-3, MeO-2
3	-	139.0	1, HO-3	-	140.1	1, HO-3 *
4	-	139.5	MeO-4, HO-3	-	139.6	MeO-4, HO-3
4a	-	123.5	1, 9	-	124.2	1
4b	-	123.6	6, 8, HO-5	-	122.5	6, 8, HO-5
5	-	151.1	7, HO-5	-	151.2	6 *,7, HO-5 *
6	6.77 (1H, d, 7.5)	116.1	8, HO-5	6.76 (1H, dd, 8.0, 1.0)	117.3	7 *,8, HO-5
7	7.13 (1H, t, 7.5)	128.2	-	6.93 (1H, t, 8.0)	128.6	6 *
8	7.05 (1H, d, 7.5)	116.0	6, 9	6.65 (1H, dd, 8.0, 1.0)	115.6	6
8a	-	148.6	7, 9 *, HO-9	-	148.8	7
9	5.38 (1H, d, 7.8)	74.5	1, 8, HO-9	-	87.4	6′, 1, 8
9a	-	137.4	9 *, HO-9	-	137.2	1 *
MeO-2	3.93 (3H, s)	56.0	-	3.77 (3H, s)	56.0	-
MeO-4	4.12 (3H, s)	61.4	-	4.18 (3H, s)	61.6	-
HO-3	7.91 (s)	-	-	8.11 (s)	-	-
HO-5	9.44 (s)	-	-	9.56 (s)	-	-
HO-9	4.57 (d, 7.8)	-	-	-	-	-
1′				6.61 (1H, s)	105.3	10′
2′				-	152.9	1′ *, MeO-2′
3′				-	137.3	1′, MeO-3′
4′				-	145.3	-
4a′				-	114.0	1′, 10′
4b′				-	120.6	6′, 9′
5′				-	123.4	6′ *
6′				6.04 (1H, s)	105.4	-
7′				-	146.5	6′*, MeO-7′, HO-8′
8′				-	143.4	6′, 9′, HO-8′ *
8a′				-	119.2	10′, HO-8′
9′				3.09 (1H, m), 2.78 (1H, m)	20.9	10′ *
10′				2.93 (2H, m)	26.9	1′, 9′ *
10a′				-	128.6	1′ *, 9′
MeO-2′				3.82 (3H, s)	55.5	-
MeO-3′				3.37 (3H, s)	59.6	-
MeO-7′				3.54 (3H, s)	55.4	-
HO-8′	-	-	-	7.61 (s)	-	-

^a 1^H (300 MHz) and ^13^C-NMR (75 MHz); ^b 1^H (500 MHz) and ^13^C-NMR (125 MHz); * two-bond coupling.

**Table 2:** Kinetic parameters of *α*-glucosidase inhibition in the presence of **2**.

Inhibitors	Dose (μM)	V*_max_* ∆OD_/_min	K*_m_* (mM)	K*_i_* (μM)
None	-	0.12	1.55	
**2**	22	0.052	1.19	20.38
	11	0.086	1.23	
Acarbose	930	0.11	4.17	190.57
	465	0.10	6.74	
